# Supplementary material for: A Possible Antioxidant Role for Vitamin D in Soccer Players: A Retrospective Analysis of Psychophysical Stress Markers in a Professional Team
Source: Int J Environ Res Public Health. 2020 May 16;17(10):3484. doi: 10.3390/ijerph17103484 (PMC7277111; doi:10.3390/ijerph17103484)
Supplement: Supplementary file 1 [file ijerph-17-03484-s001.pdf]

International Journal of
Environmental Research and Public Health

MDPI

Supplementary Material

Table 1. VitD levels (ng/mL) measured during the soccer season 2017/2018. Light grey cells represent insufficient/deficient levels (<30 ng/mL). Dark grey cells represent deficient vitD levels (<20 ng/mL). Red numbers indicate that the corresponding soccer player was on an injury period. The last two rows represents the average vitD values and the corresponding standard deviations.

	05.07.17	13.09.17	29.11.17	15.01.18	06.03.18	24.04.18	10.07.18	15.08.18
**Player1**	24.4	25.2	23	26.1	23	26.9	27.7	28.8
**Player2**	42.1	35.3	31.3	21	24.9	23.4	50.3	36.3
**Player3**	62.4	50.2	36.9	**38.1**	**28.8**	33.5		
**Player4**	25.9	25.4	23	27.2			33.2	30.7
**Player5**	23.1	27.8	26.6	**18.6**				
**Player6**	**53.6**	32.4	**23.3**	18.8	**18.7**	23.9	68.3	36.4
**Player7**	29	23.5	21.3	24.7	**24.1**	26.7	38.8	
**Player8**	65.1	40.7	40.4	45.6	32.7	28.4	55	
**Player9**	34.1	30.3	**39.2**	20.7	21.4	26.2	52.7	32.7
**Player10**	**26.6**	**28.4**		**18**	**21.4**		**52.6**	**32.8**
**Player11**	22	28.3	25.9	17.7	19	24.2	23.5	
**Player12**	52.9	42	37.1	36.7	29.5	32.8	55.2	36.3
**Player13**	44.6	41	41.9	37.8	38.6	35.3		40.3
**Player14**	**28.8**	31.2	24.6	16.3	18.1	**23.4**	50.1	35.5
**Player15**	18.1	17.3	20.2	14.7	20.4	15.7		19.9
**Player16**			17.6	17.1	11.7			
**Player17**		23.1	19.5	26	22.9	**27.4**		34.7
**Player18**	36.9	**33**	31.5	33.2	24.7	32.8	42.1	35.6
**Player19**	35.2			22.7	25.4	**29.7**	41.5	33.8
**Player20**	18.9	12.9	13.6	9.1	7.9		19.8	15.9
**Player21**	27.7	29.9	25.7	17.7	22.1	27.1	47.9	
**Player22**	24.4		**26**	**28.7**	29	35.6	28.6	35.3
**Player23**	34.2	38.3	35.4	31.9	24.8	27.6	41	40.1
**Player24**			11.3	14.1	5.7			
**Player25**	49.2	35.2	33	23.9	22.5	26.7		
**Player26**		42.5	37.5	33.2	23.5	31.6	65.2	43.3
**Player27**	38.9	34.6	29.9	25.4	24.6	32.6	52.6	36.6
**Player28**		24.7	29.1	20.5	21.7	23.5	**27.7**	
**Player29**	60.5	37.4	34	36.6	25.5	29.7	51.3	34.8
**Average**	36.6	31.6	28.1	24.9	22.7	28.0	44.1	33.7
**STD**	14.2	8.5	8.2	8.8	6.8	4.7	13.4	6.5

Table 2. CK levels (U/L) measured during the soccer season 2017/2018. Grey cells represents the levels exceeding the 195 U/L normal clinical limit. Percentages in the third to last row indicate the fraction of measurements exceeding the 195 U/L limit. Red numbers indicate that the corresponding soccer player was on an injury period. The last two rows represents the average CK values and the corresponding standard deviations.

	05.07.17	13.09.17	29.11.17	15.01.18	06.03.18	24.04.18	10.07.18	15.08.18
**Player1**	233	219	217	227	321	213	518	269
**Player2**	162	249	171	292	327	161	404	572
**Player3**	214	294	**296**	**143**	508	195		
**Player4**	191	488	433	289			400	361
**Player5**	420	260	229	**286**				
**Player6**	**692**	791	**454**	662	**339**	659	842	286
**Player7**	537	215	233	265	**148**	192	391	
**Player8**	202	155	246	138	171	162	377	
**Player9**	906	156	**203**	184	180	206	182	327
**Player10**	**217**	**185**		**234**	**274**		**367**	**194**
**Player11**	509	292	283	297	292	254	1195	
**Player12**	400	222	208	326	210	266	1240	357
**Player13**	520	236	447	163	181	211		257
**Player14**	**325**	257	491	177	228	**180**	315	263
**Player15**	283	455	1479	301	382	968		256
**Player16**			552	232	582			
**Player17**		348	419	197	256	**114**		444
**Player18**	303	**678**	207	156	175	194	454	535
**Player19**	275			457	355	**289**	717	403
**Player20**	242	678	649	757	413		2018	158
**Player21**	164	140	128	103	129	236	167	
**Player22**	176	290	**183**	**115**	219	225	393	234
**Player23**	247	107	253	185	163	106	341	92
**Player24**			754	476	586			
**Player25**	153	349	626	563	1209	620		
**Player26**		326	610	294	346	296	298	321
**Player27**	272	269	628	168	300	373	624	271
**Player28**		74	319	121	99	149	**249**	
**Player29**	343	200	257	115	140	124	299	296
**>195 U/L**	79.2 %	76.9%	88.9%	58.7%	66.6%	56.6%	90.5%	89.5%
**Average**	332.7	305.1	406.5	273.2	316.0	277.9	561.5	310.3
**STD**	185.7	179.0	278.0	164.7	221.2	204.7	443.2	118.5

Table 3. ROS levels (Car/U) measured during the soccer season 2017/2018. Grey cells represents the levels exceeding the 300 Car/U normal clinical limit. Percentages in the third Table 300. Car/U limit. Red numbers indicate that the corresponding soccer player was on an injury period. The last two rows represents the average ROS values and the corresponding standard deviations.

	05.07.17	13.09.17	29.11.17	15.01.18	06.03.18	24.04.18	10.07.18	15.08.18
**Player1**	240	233	271	303	301	223	282	258
**Player2**	313	332	322	372	348	249	311	359
**Player3**	231	291	237	312	293	226		
**Player4**	320	301	377	368			266	261
**Player5**	280	247	279	275				
**Player6**	271	385	300	308	247	230	310	250
**Player7**	337	309	393	331	371	204	283	
**Player8**	187	225	259	343	294	213	296	
**Player9**	283	310	272	272	362	228	278	317
**Player10**	192	244		211	220		185	180
**Player11**	359	379	306	348	377	265	302	
**Player12**	345	372	409	353	363	258	315	314
**Player13**	199	258	341	258	281	179		239
**Player14**	267	334	411	382	321	203	315	293
**Player15**	342	373	383	381	350	254		288
**Player16**			301	300	299			
**Player17**		235	281	290	294	169		222
**Player18**	224	267	354	316	390	236	223	261
**Player19**	270			291	336	210	283	329
**Player20**	336	301	272	311	301		279	244
**Player21**	313	389	347	359	375	296	301	
**Player22**	286	319	272	303	275	215	283	291
**Player23**	212	269	296	356	319	193	250	226
**Player24**			324	434	358			
**Player25**	263	287	383	302	276	219		
**Player26**		299	306	337	292	211	260	262
**Player27**	257	300	297	341	318	251	267	253
**Player28**		231	318	294	355	193	220	
**Player29**	352	296	313	281	309	234	251	261
**>300 Car/U**	37.5%	50.0%	59.2%	72.4%	63.0%	0%	28.6%	21.1%
**Average**	278.3	299.5	319.4	321.8	319.4	224.3	274.2	270.2
**STD**	53.4	50.5	48.2	45.2	42.5	29.4	34.0	42.9

Table 4. T/C ratios measured during the soccer season 2017/2018. Value in grey cells represents T/C ratio consistent with an overtraining risk (<0.76). Red numbers indicate that the corresponding soccer player was on an injury period. The last two rows represents the average T/C ratio and the corresponding standard deviation.

	05.07.17	13.09.17	29.11.17	15.01.18	06.03.18	24.04.18	10.07.18	15.08.18
**Player1**	2.17	1.69	1.89	1.77	1.60	1.69	1.61	1.33
**Player2**	1.05	1.02	1.40	1.16	0.89	1.13	0.71	0.92
**Player3**	1.03	0.78	**0.83**	**1.36**	1.36	1.19		
**Player4**	1.32	1.07	1.51	1.26			1.00	0.96
**Player5**	1.96	0.98	1.14	**1.04**				
**Player6**	**1.14**	0.94	**1.07**	0.99	**1.00**	1.14	0.96	0.98
**Player7**	0.95	0.88	1.33	1.64	**1.07**	1.30	0.90	
**Player8**	0.46	1.17	1.32	1.10	1.27	1.48	0.74	
**Player9**	0.68	0.67	**1.15**	0.60	0.82	0.81	1.03	0.81
**Player10**	**0.97**	**1.09**		**1.15**	**0.79**		**1.65**	**1.26**
**Player11**	0.75	0.84	1.62	2.71	1.18	0.94	0.74	
**Player12**	1.27	1.61	1.32	1.59	0.88	0.87	0.82	1.58
**Player13**	1.23	1.21	1.18	1.92	0.84	1.02		1.13
**Player14**	**1.40**	1.47	1.02	1.34	0.93	**1.62**	0.74	1.53
**Player15**	2.31	1.04	1.26	1.79	1.07	1.08		1.98
**Player16**			1.12	1.03	0.91			
**Player17**		1.40	1.06	1.34	0.88	**1.34**		1.02
**Player18**	0.91	**1.09**	0.80	1.01	0.86	0.94	1.08	0.99
**Player19**	0.90			0.91	1.06	**1.13**	0.98	1.37
**Player20**	5.98	1.27	1.28	1.15	1.28		1.16	3.72
**Player21**	0.95	1.28	1.22	1.35	1.62	1.17	0.99	
**Player22**	3.18	1.00	**1.11**	**1.06**	0.92	1.02	1.20	1.36
**Player23**	1.95	1.69	1.76	2.58	1.46	1.58	1.27	1.42
**Player24**			2.07	1.93	2.12			
**Player25**	1.73	1.07	1.08	1.14	0.92	0.96		
**Player26**		1.35	1.31	1.64	1.36	1.26	1.35	1.70
**Player27**	1.66	1.70	1.18	1.30	1.08	0.92	1.50	1.59
**Player28**		0.64	1.04	0.73	0.51	1.05	**0.65**	
**Player29**	0.81	0.91	1.24	1.62	0.94	1.09	1.48	0.99
**Average**	1.53	1.15	1.27	1.39	1.10	1.16	1.07	1.40
**STD**	1.13	0.30	0.29	0.48	0.33	0.24	0.30	0.64
